# Near-Infrared Laser Photobiomodulation Reduces Pro-Inflammatory Cytokines in an In Vitro Model of Bronchopulmonary Dysplasia: A Preliminary Report

**DOI:** 10.3390/medsci14010152

**Published:** 2026-03-20

**Authors:** Carlo Dani, Camilla Fazi, Francesca Cialdai, Chiara Risaliti, Lorenzo Notari, Monica Monici

**Affiliations:** 1 Department of Neurosciences, Psychology, Drug Research and Child Health, University of Florence, 50139 Florence, Italy; camilla.fazi@unifi.it; 2 Division of Neonatology, Careggi University Hospital of Florence, 50134 Florence, Italy; 3 ASA Campus Joint Laboratory, ASA Research Division, Department of Experimental and Clinical Biomedical Sciences “Mario Serio”, University of Florence, 50139 Florence, Italy

**Keywords:** photobiomodulation, laser, lung slice, preterm rabbit, bronchopulmonary dysplasia

## Abstract

**Background:** The multifactorial pathogenesis of bronchopulmonary dysplasia (BPD) includes prematurity, inflammation, and oxidative stress. Photobiomodulation therapy (PBMT) using near-infrared (NIR) laser sources was found to have anti-inflammatory effects in several respiratory disorders. Our aim was to evaluate whether PBMT could reduce inflammation in an in vitro model of BPD. **Materials and Methods:** Precision-cut lung slices (PCLSs) from premature rabbits were exposed to lipopolysaccharide (LPS) and treated with three PBMT protocols (A, B, and C) differing for the treatment parameter such as fluence (energy delivered per unit area, laser A: 7.09, laser B: 7.41, laser C: 7.01 J/cm^2^) and exposure time (25, 20, 12 s, respectively). The expression level of TNFα and IL-6 was measured by reverse transcription quantitative polymerase chain reaction (RT-qPCR) after 2 or 6 h from PBMT. **Results:** PBMT protocols A and B reduced IL-6 and TNFα mRNA at both timepoints, although the effect was less pronounced after 6 h than after 2 h. Furthermore, protocol A, which involved intermediate fluence and longer laser exposure, was more effective than protocol B. **Conclusions:** PBMT with NIR laser sources has an effective anti-inflammatory effect in an in vitro model of BPD, such as PCLSs from premature rabbits pretreated with LPS. These encouraging results support the planning of further studies in animal models of BPD and help identify the most effective PBMT protocol to use.

## 1. Introduction

Despite the relevant advances of perinatal assistance over the past 30 years, bronchopulmonary dysplasia (BPD) still remains one of the most severe complications of prematurity, and its prevalence is increasing as a consequence of the increased survival of very preterm infants [1,2]. Premature delivery is the main risk factor for BPD and, in fact, approximately 80% of infants born at 22–24 weeks of gestation develop BPD, while this percentage decreases to 20% in infants born at 28 weeks of gestational age [3,4,5].

BPD involves reduced pulmonary alveolarization and abnormal vascular development that induce different degrees of respiratory failure and different phenotypes, such as BPD with moderate to severe parenchymal disease, pulmonary hypertension, or large airway disease [6].

The multifactorial pathogenesis of BPD includes inflammation and oxidative stress that damage the premature lungs and are induced by barotrauma, volutrauma, and oxygen therapy due to respiratory support and mainly mechanical ventilation [7]. Indeed, tracheal aspirates and lung lavages from premature infants with BPD show high levels of pro-inflammatory cytokines, chemokines, soluble adhesion molecules, and growth factors related to neutrophil- and monocyte/macrophage-mediated inflammation [8,9,10,11,12,13,14].

On the other hand, to date, the only drugs effective in the prevention and treatment of BPD, namely, postnatal corticosteroids, were associated with an increased risk of neurodevelopmental impairment [15]. Therefore, the availability of new and effective treatments for BPD, with a high safety profile, is urgently needed to improve outcomes in preterm infants.

Pulmonary photobiomodulation therapy (PBMT) using different laser sources was used to treat several respiratory disorders, such as pneumonia, asthma, and chronic obstructive pulmonary disease (COPD) in children, adults, and elderly patients [16,17,18,19,20]. It showed clinical benefits, since it reduced recovery time, need for medications, and respiratory symptoms and improved radiological, immunological, and blood parameters [16,17,18,19,20]. PBMT uses non-ionizing light sources in the visible and infrared spectra (600–1200 nm), which reduce inflammation and stimulate healing [21]. It was found in animal models to reduce pulmonary microvascular leakage, activate macrophage, T-cell, and neutrophil influx, reduce pro-inflammatory interleukins, such as interkeukin-1β (IL-1β), interleukin-6 (IL-6), and tumor necrosis factor α (TNFα), increase anti-inflammatory cytokines, such as interleukin 10 (IL-10), and reduce collagen deposition [22,23,24,25,26,27,28].

Based on previous considerations [16,17,18,19,20,21,22,23,24,25,26,27,28], we hypothesized that PBMT may decrease inflammation in an in vitro model of BPD. To evaluate this hypothesis, we conducted this proof-of-concept study in which inflammatory biomarkers were measured in precision-cut lung slices (PCLSs) from premature rabbits exposed to lipopolysaccharide (LPS) and treated or untreated with PBMT using a near-infrared (NIR) laser source.

## 2. Materials and Methods

In this study, we compared the effects of exposing precision-cut lung slices (PCLSs), previously incubated with LPS to mimic BPD-associated inflammation, to three different NIR laser treatments versus unexposed PCLSs. Lipopolysaccharide (LPS) was used as a pro-inflammatory stimulus to mimic the inflammatory environment characteristic of BPD [29]. LPS activates macrophages and epithelial cells, inducing the production of key cytokines, such as IL-6 and TNFα, which are central mediators of inflammation in preterm lungs.

**Figure 1:**
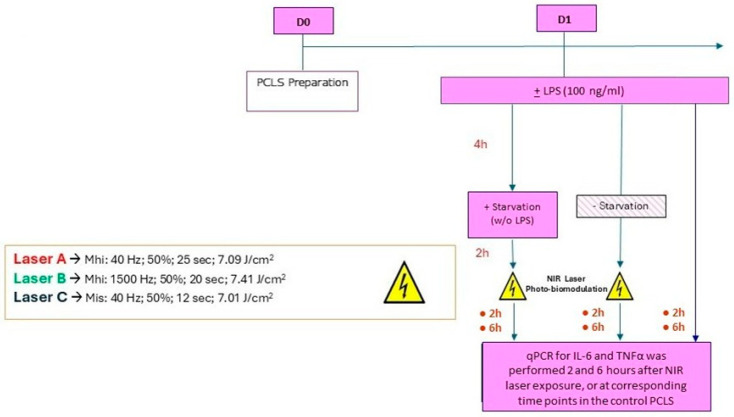
Day 0 (D0) indicates the day of precision-cut lung slice (PCLS) preparation. On day 1 (D1), PCLSs were exposed to 100 ng/mL of lipopolysaccharide (LPS) for 4 h to induce inflammation. After 4 h, a portion of the PCLS was deprived of LPS (starvation) prior to near-infrared (NIR) laser photobiomodulation therapy (PBMT). NIR laser treatments were performed according to protocols A, B, and C. Expression levels of TNFα and IL-6 were measured at 2 and 6 h after NIR laser exposure or at corresponding timepoints in the control PCLS.

Specifically, we evaluated the potential anti-inflammatory effects of the NIR laser treatments by measuring and comparing the expression of tumor necrosis factor (TNFα) and interleukin-6 (IL-6) in both exposed and unexposed PCLSs. The study design is summarized in Figure 1.

### 2.1. Experimental Animals

Time-mated New Zealand White rabbits were obtained from Charles River Laboratories (France). Pregnancy was confirmed with ultrasounds at 12–14 days post-artificial insemination, and they were then housed (temperature 15–21 °C, relative humidity 55 ± 15%, 12:12 h light/dark cycles, with food and water ad libitum) at Chiesi’s animal facility until C-section. The experimental procedure was approved by the local animal ethics committee and met the standard European regulations on animal research (n° 875/2021-PR).

The experiments were performed as outlined in Figure 1. Each experiment was conducted only once due to extremely limited resources.

### 2.2. PCLS Preparation

Three preterm rabbit pups, delivered by caesarean section on day 28 of gestation, were used for the preparation of PCLSs. From each caudal lobe of each pup, a mean number of 25–30 PCLSs was obtained from the agarose-inflated lung, following the experimental procedure described by Ragionieri et al. [30]. PCLSs that were 300 μm thick were cut with a vibratome (Leica VT1200 S, Leica Biosystem, Buccinasco, Italy). After that, these slices were further processed to obtain from each of them round portions 5 mm in diameter named “punches,” which were made manually with the aid of a biopsy punch (Kai Industries Co., Seki, Japan). PCLSs were placed into 24-well plates: 4 punches/well with 0.6 mL/well of SF-DMEM/F-12 (Gibco, Grand Island, NY, USA) medium enriched with antibiotics (P/S, Invitrogene, Carlsbad, CA, USA) and antimycotic (AmphB, Merck-Sigma, St. Louis, MO, USA). The plates were incubated in custom humidified incubators (Okolab, Pozzuoli, Italy) at 37 °C, with 5% CO_2_ and 21% O_2_. A constant flow of compressed air (0.8 L/min) was maintained within the incubators to avoid excessive humidity buildup. The day of PCLS preparation (cutting) was indicated as day 0. During day 0, media were changed three times to remove agarose residuals and to prevent microbial contamination. On day 1, 100 ng/mL of lipopolysaccharide (LPS, Cat Nr L4391_O111:B4, Sigma-Aldrich, St. Luis, MO, USA) was added to induce the inflammatory environment in vitro. After 4 h, a part of the PCLS was deliberately deprived of LPS exposure (starvation) before laser treatment. This was done to prevent prolonged exposure to LPS from causing inflammation too severe to be reduced by PBMT.

We selected a 4 h LPS exposure based on well-established kinetics of early pro-inflammatory gene activation, where transcriptional upregulation of TNFα and IL-6 typically peaks between 2 and 4 h following TLR4 stimulation by LPS [31].

### 2.3. NIR Laser Treatment in PCLSs

NIR laser treatment in PCLSs was performed with Multiwave Locked Systems (MLS^®^ laser systems, ASA S.r.l., Vicenza, Italy) that combine two NIR laser sources with simultaneous and synchronized emission. MLS is a family of class IIIb and IV diode lasers equipped with a source with continuous light emission at 808 nm (power from 100 mW to 6 W) that can work in frequency mode (frequency from 1–2000 Hz) and a second source that operates in pulsed mode at 905 nm, with peak power from 25 W to 1 KW and frequency of the pulse trains that can be synchronized with that of the 808 nm source. Within each pulse train, the frequency can vary from 10 KHz to 90 KHz.

PCLSs were exposed once to NIR laser treatment immediately after 4 h of LPS exposure using three treatment protocols (A, B, C) to evaluate the efficacy of different energy delivery modes:

(A) Frequency 40 Hz, intensity 50%, energy density 7.09 J/cm^2^, exposure time 25 s, mean power 850 mW, and power density 283 mW/cm^2^ (MLS^®^-MHi, ASA Srl., Vicenza, Italy);
(B) Frequency 1500 Hz, intensity 50%, 7.41 J/cm^2^, exposure time 20 s, mean power 1.1 W, and power density 366 mW/cm^2^ (MLS^®^-MHi, ASA Srl., Vicenza, Italy);
(C) Frequency 40 Hz, intensity 50%, energy density 7.01 J/cm^2^, exposure time 11 s, mean power 1.91 W, and power density 636 mW/cm^2^ (MLS-MiS, ASA Srl., Vicenza, Italy).

Energy density, or fluence, is the energy delivered per unit area, while power density is the amount of power per unit area. The three protocols have been designed to have very similar fluences and allow the energy delivery mode to be tested. A fluence value around 7 J/cm^2^ has been chosen on the basis of previous studies, both in vitro and in vivo, in which MLS laser sources have been applied.

The laser spot size was 3 cm^2^. PCLSs have a roughly elliptical shape, with the major axis measuring approximately 3 mm, and the minor axis approximately 1.5 mm. For laser treatment, each slice was positioned at the center of the spot, with the handpiece held, using a suitable holder, in a fixed position perpendicular to the sample.

### 2.4. Reverse Transcription Quantitative Polymerase Chain Reaction (RT-qPCR)

RT-qPCR was used to detect the expression levels of tumor necrosis factor (TNFα) and interleukin-6 (IL-6). RNA was isolated using the RNeasy Mini kit (Qiagen, Hilden, Germany) according to the manufacturer’s instructions. Total RNA was extracted from precision-cut lung slices (PCLSs), and complementary DNA (cDNA) was synthesized from 250 ng of total RNA using SuperScript IV VILO reverse transcriptase (Thermo Fisher Scientific, Waltham, MA, USA), according to the manufacturer’s instructions. One microliter of the cDNA mixture was used for real-time PCR experiments to measure the levels of IL-6 (Oc04097053, Thermo Fisher Scientific, Waltham, MA, USA) and TNF-α (Oc03397715, Thermo Fisher Scientific, Waltham, MA, USA). Differences in gene expression levels were determined by the 2^−ΔCt^ formula using VINCULIN Oc06749903 (Thermo Fisher Scientific, Waltham, MA, USA) as a housekeeping gene. No external calibrator was used; values represent expression levels relative to VINCULIN. Real-time PCR reactions were conducted on a StepOnePlus™ Real-Time PCR System (Thermo Fisher Scientific, Waltham, MA, USA) using TaqMan Fast Advanced Master Mix (Thermo Fisher Scientific, Waltham, MA, USA) according to the manufacturer’s protocol.

The expression level of TNFα and IL-6 was measured after 2 or 6 h from NIR laser exposure.

### 2.5. Statistical Analysis

The primary endpoint of the study was to evaluate the effect of NIR laser treatment of PCLSs on the expression of TNFα and IL-6 compared to controls.

All data were presented as mean ± SD. Data were analyzed with one-way ANOVA followed by Dunnett’s multiple comparisons test. Statistical analysis was performed using GraphPad Prism 10.4 software. A value of *p* < 0.05 was considered statistically significant.

## 3. Results

At 6 and 10 h after LPS exposure, a significant increase in the expression of the pro-inflammatory cytokines IL-6 and TNFα was observed in PCLSs. As expected, cytokine expression was higher 6 h after laser application than 2 h after.

### 3.1. Changes in IL-6 and TNFα 2 Hours After Laser Treatment

**Figure 2:**
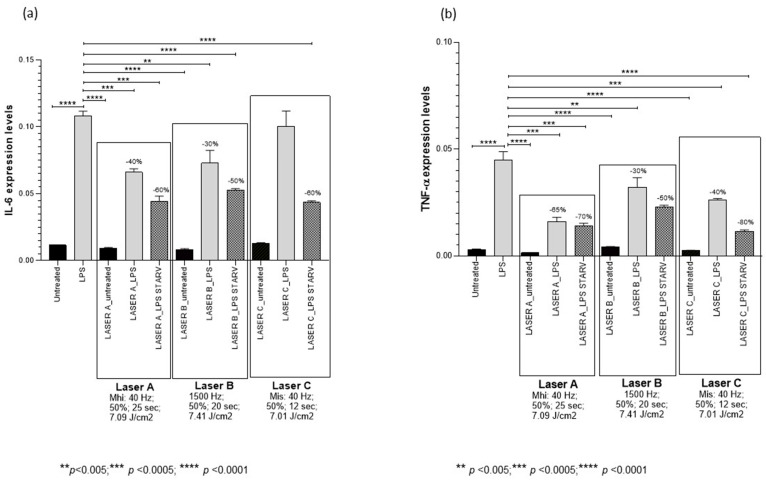
Changes in IL-6 (panel **a**) and TNF-α (panel **b**) mRNA expression levels in precision-cut lung slices (PCLSs) untreated or treated for 4 h with lipopolysaccharide (LPS), unexposed or exposed to NIR laser, with or without starvation. Gene expression was assessed 2 h after laser application. In control PCLSs, mRNA levels were measured at the corresponding timepoint (i.e., 2 h after the time at which laser treatment would have been applied). Two hours after treatment, NIR laser protocols A and B significantly decreased IL-6 and TNF-α mRNA levels in both LPS-treated and LPS-starved PCLSs. In contrast, protocol C had no effect on IL-6 mRNA in LPS-treated PCLSs but reduced it in LPS-starved PCLSs, while it decreased TNF-α mRNA in both LPS-treated and LPS-starved PCLSs. Means ± SDs.

PBMT protocol A decreased IL-6 mRNA by 40% (*p* < 0.0001) in LPS-treated PCLSs and by 60% (*p* < 0.0001) in LPS-starved PCLSs compared to controls. Moreover, it decreased TNFα mRNA by 65% (*p* < 0.0005) in LPS-treated PCLSs and by 70% (*p* < 0.0005) in LPS-starved PCLSs compared to controls (Figure 2, Table 1).

PBMT protocol B decreased IL-6 mRNA by 30% (*p* < 0.005) in LPS-treated PCLSs and by 50% (*p* < 0.0005) in LPS-starved PCLSs compared to controls. Moreover, it decreased TNFα mRNA by 30% (*p* < 0.005) in LPS-treated PCLSs and by 50% (*p* < 0.0005) in LPS-starved PCLSs compared to controls (Figure 2, Table 1).

PBMT protocol C had no effect on IL-6 mRNA in LPS-treated PCLSs and decreased it by 60% (*p* < 0.0005) in LPS-starved PCLSs compared to controls. Moreover, it decreased TNFα mRNA by 40% (*p* < 0.0005) in LPS-treated PCLSs and by 80% (*p* < 0.0001) in LPS-starved PCLSs compared to controls (Figure 2, Table 1).

### 3.2. Changes in IL-6 and TNFα 6 Hours After Laser Treatment

**Figure 3:**
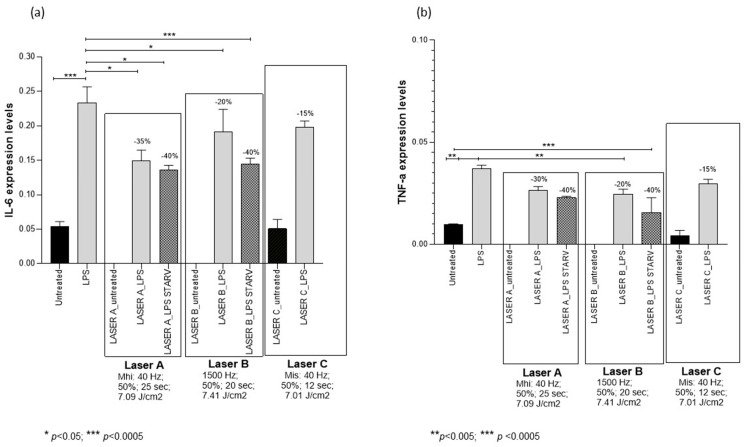
Changes in IL-6 (panel **a**) and TNF-α (panel **b**) mRNA expression levels in precision-cut lung slices (PCLSs) untreated or treated for 4 h with lipopolysaccharide (LPS), unexposed or exposed to NIR laser (protocols A, B, or C), with or without starvation. Gene expression was assessed 6 h after laser application. In control PCLSs, mRNA levels were measured at the corresponding timepoint (i.e., 6 h after the time at which laser treatment would have been applied). Six hours after treatment, protocols A and B significantly decreased IL-6 mRNA expression in both LPS-treated and LPS-starved PCLSs, while reductions in TNF-α mRNA were observed but were not consistently statistically significant. Protocol C did not significantly affect IL-6 or TNF-α mRNA expression at this timepoint. Means ± SDs.

PBMT protocol A decreased IL-6 mRNA by 35% (*p* < 0.05) in LPS-treated PCLSs and by 40% (*p* < 0.05) in LPS-starved PCLSs compared to controls. Moreover, it decreased TNFα mRNA by 30% in LPS-treated PCLSs and by 40% in LPS-starved PCLSs compared to controls, although these decreases were not statistically significant (Figure 3, Table 2).

PBMT protocol B decreased IL-6 mRNA by 20% (*p* < 0.05) in LPS-treated PCLSs and by 40% (*p* < 0.0005) in LPS-starved PCLSs compared to controls. Moreover, it significantly decreased TNFα mRNA by 20% (*p* < 0.005) in LPS-treated PCLSs and by 40% (*p* < 0.0005) in LPS-starved PCLSs compared to controls (Figure 3, Table 2).

Laser C exposure did not affect IL-6 and TNFα mRNA expression (Figure 3, Table 2).

## 4. Discussion

In this study, we evaluated for the first time whether PBMT by NIR laser treatment could reduce inflammation in an in vitro model of BPD. We exposed PCLSs from premature rabbits incubated with LPS to three different protocols of NIR laser treatment (A, B, or C) characterized by the same emission wavelengths (808 nm and 904 nm, simultaneous and synchronized) and intensity (50%), similar fluences (energy delivered per unit area, 7.09, 7.41, and 7.01) that were delivered with different exposure times according to the different power densities. We found that laser protocols A and B significantly reduced IL-6 and TNFα mRNA both 2 and 6 h after laser treatment. As expected, these effects were more pronounced in starved PCLSs due to the shorter LPS exposure, while in non-starved PCLSs, the effects were less pronounced at 6 h compared to 2 h, likely due to the longer LPS exposure. Moreover, the laser protocol A was more effective than protocol B in providing a decrease in IL-6 and TNFα mRNA at both timepoints. In contrast, protocol laser C was less effective than lasers A and B, as it only reduced TNFα mRNA expression 2 h after treatment and was not effective 6 h after treatment. Overall, protocol laser A, which had an intermediate fluence, the lowest power density, and the longest exposure time, was shown to have the greatest anti-inflammatory effect. These results indicate that, as expected, the small differences in energy density between the three protocols used did not lead to significant changes in efficacy. In fact, the most pronounced anti-inflammatory effect was obtained with the intermediate fluence (protocol A, 7.09 J/cm), and the weakest anti-inflammatory effect was obtained with protocol C, which had a fluence closer to protocol A than to protocol B. Frequency also appeared to have little influence on the treatment effect, as protocols A and C, which had the same frequency (40 Hz), showed the greatest and least anti-inflammatory effect, respectively, while the efficacy of protocol B (1500 Hz) was intermediate.

Interestingly, the magnitude of the anti-inflammatory effect increased with decreasing power density (protocol A 283 mW/cm^2^, protocol B 366 mW/cm^2^, protocol C = 636 mW/cm^2^) and with increasing exposure time (protocol A 25 s, protocol B 20 s, protocol C 11 s), suggesting that the anti-inflammatory effect is also determined by the method of energy administration: the same energy administered for longer times and with a lower power density was more effective. These findings are consistent with the well-described biphasic dose–response pattern of PBMT [21]. According to this principle, biological responses are influenced not only by total energy (fluence) but also by the mode of energy delivery. Lower irradiance applied over a longer duration may allow more controlled mitochondrial photostimulation, particularly at the level of cytochrome c oxidase, resulting in moderate reactive oxygen species (ROS) generation and optimized activation of redox-sensitive signaling pathways. This controlled signaling can promote anti-inflammatory gene modulation without triggering cellular stress responses that may occur at higher power densities.

Our findings are important because IL-6 and TNFα mRNA were both found to be implicated in the inflammatory pathogenesis of BPD. Kotecha et al. reported that IL-6 expression was nearly tenfold higher in the bronchoalveolar lavage fluid of nine extremely preterm infants who developed BPD compared with controls and that it was expressed primarily by alveolar macrophages [8]. Jonsson et al. confirmed these results. They demonstrated in 17 preterm infants who developed BPD that TNFα concentration in the bronchoalveolar lavage fluid increased by days 6 and 7 compared to controls, while IL-6 increased by days 2 and 3 [11]. These studies are very relevant, since the role of IL-6 in the pathogenesis of BPD was recently emphasized by Hirani et al. in a mouse model of BPD [32]. They presented a novel IL-6-mediated mechanism by which hyperoxia activates macrophages in immature lungs, impairs alveolar epithelial type II homeostasis, and disrupts elastic fibre formation, thereby inhibiting lung growth [32]. The data provide evidence that IL-6 trans-signaling could offer an innovative pharmacological target to enable lung growth in severe BPD.

Therefore, it is of great interest that PBMT was found to decrease IL-6 and TNFα in several animal models of lung pathologies. De Brito et al. reported that PBMT downregulated pro-inflammatory cytokine, such as IL-1β, IL-6, and TNFα, and upregulated the anti-inflammatory IL-10 in a murine model of idiopathic pulmonary fibrosis [24]; da Cunha Moraes et al. found that PBMT reduced the number of inflammatory cells and the pro-inflammatory cytokine secretion, such as IL-1β, IL-6, and TNFα, in bronchoalveolar lavage fluid in a murine model of chronic obstructive pulmonary disease (COPD) [26]; and Miranda da Silva et al. showed that PBMT reduced lung levels of IL-6 and TNF-α and increased levels of IL-10 in the rat model of lung inflammation induced by a pollutant [27]. Oliveira et al. demonstrated that PBMT reduced pulmonary inflammation and levels of IL-1β, IL-6, and TNFα in bronchoalveolar lavage fluid in a murine model of acute respiratory distress syndrome [28]. While the anti-inflammatory effect of PBMT, when applied to lung pathologies, is confirmed by these studies [24,26,27,28], the action mechanisms underlying the biological response have not yet been sufficiently explored in the various studies. It is well known that the mechanisms leading to the biological response strongly depend on the laser emission wavelength and its absorption by the endogenous chromophores of tissues. Therefore, when comparing PBMT studies, the emission wavelength of the sources used should be carefully considered. As for the PBMT by sources emitting in the red region of the electromagnetic spectrum (wavelengths approximately between 620 and 750), it has been suggested that the anti-inflammatory effects of PBMT are likely due to the downregulation of the purinergic receptor (P2X7r) expression, which reduces pro-inflammatory cytokines, increases anti-inflammatory cytokines, and decreases collagen deposition [22]. On the other hand, we used a dual-wavelength laser system whose anti-inflammatory effect was demonstrated in in vitro studies [33,34,35], in animal models [36,37], and human patients affected by SARS-CoV-2 infection [20]. Proteomic studies showed that PMBT with dual-wavelength laser systems increases the synthesis of NLRP10, a member of the NOD-like receptor family of cytoplasmic receptors, which can downregulate the inflammatory response [38] by inhibiting caspase 1 activity and reducing IL-1β and interleukin-18 (IL-18) production [39,40]. Both these pro-inflammatory cytokines play a key role in inducing IL-6 and TNF-α [41,42,43]. Therefore, the decrease in IL-6 and TNF-α levels induced by PBMT in our PCLS model of BPD may have been caused by the decrease in IL-1β and IL-18 induced by the increase in nucleotide-binding domain leucine-rich repeat-containing receptor-10 (NLRP10). However, further studies are needed to prove this hypothesis.

In summary, our findings in this model of BPD suggest that PBMT with an NIR laser could represent an anti-inflammatory, promising therapy for BPD due to its ability to reduce IL-6 and TNFα expression. This is particularly important considering that over the last 30 years, the treatment of BPD has not changed and has remained essentially based on the use of corticosteroids.

This study has several limitations. Due to limited resources, some experiments at 6 h could not be repeated because of insufficient mRNA quality. This occurred in the control PCLS of the laser groups for protocols A and B and in the starved PCLS of the laser group for protocol C. The small number of animals used may introduce a potential litter effect and limit generalizability, although multiple PCLSs per animal were randomly allocated to experimental groups. Only two pro-inflammatory cytokines (IL-6 and TNFα) were evaluated, but the evaluation of these two central markers allowed us to demonstrate that PBMT exerts a measurable anti-inflammatory effect. Only mRNA expression was evaluated, without assessment of protein levels, although transcriptional changes provide an early and sensitive indication of inflammatory modulation. Moreover, PCLSs preserve native lung architecture and cellular heterogeneity, but they do not fully reproduce the complexity of the in vivo lung and the anti-inflammatory effects observed. Therefore, the results should be interpreted as preliminary and hypothesis-generating. Nevertheless, given the consistency of the effects across treatment conditions, we believe these results provide a robust preliminary indication of the anti-inflammatory potential of PBMT in this in vitro model, which warrants confirmation in appropriate in vivo models of BPD.

Although this study evaluated ex vivo lung tissue, translation to human lungs presents several challenges. Differences in tissue optical properties and penetration depth require careful dosimetry and optimization, and application in neonates would necessitate dedicated delivery systems and rigorous safety assessment. Nevertheless, the established safety profile and non-invasive nature of PBMT [16,17,18,19,20] support further investigation in appropriate animal models before controlled clinical studies.

## 5. Conclusions

We found that PBMT with the NIR laser had an effective anti-inflammatory effect in an in vitro model of BPD, such as PCLSs from premature rabbits pretreated with LPS. The most effective laser protocol was the one with intermediate fluence and prolonged exposure, and this effect was most pronounced 2 h after treatment. These encouraging results support the planning of further studies in animal models and help identify the most effective laser treatment protocol in the hope that they can support further safety and efficacy studies in preterm infants with BPD.

## Figures and Tables

**Table 1:** IL-6 and TNF-α mRNA expression levels in precision-cut lung slices (PCLSs) untreated or treated for 4 h with lipopolysaccharide (LPS), unexposed or exposed to NIR laser protocols A, B, or C, with or without starvation, and analyzed 2 h after NIR laser treatment. In control PCLSs, mRNA expression was quantified at the corresponding timepoint (i.e., 2 h after the time at which laser treatment would have been applied). Means ± SDs.

**IL-6**										
**Controls**		**Protocol A**			**Protocol B**			**Protocol C**		
Untreated	LPS	Untreated	LPS	LPS starvation	Untreated	LPS	LPS starvation	Untreated	LPS	LPS Starvation
0.0117 ± 0.00004	0.1083 ± 0.00488	0.0093 ± 0.0009	0.0662 ± 0.0036	0.0445 ± 0.0051	0.0084 ± 0.0084	0.0729 ± 0.0729	0.0526 ± 0.0016	0.0130 ± 0.0007	0.1002 ± 0.0165	0.0437 ± 0.0016
**TNF-α**										
**Controls**		**Protocol A**			**Protocol B**			**Protocol C**		
Untreated	LPS	Untreated	LPS	LPS starvation	Untreated	LPS	LPS starvation	Untreated	LPS	LPS Starvation
0.0031 ± 0.0002	0.0450 ± 0.0054	0.0015 ± 0.0002	0.0162 ± 0.0028	0.0140 ± 0.0018	0.0041 ± 0.0005	0.0321 ± 0.0064	0.0228 ± 0.0014	0.0026 ± 0.0001	0.0264 ± 0.0007	0.0116 ± 0.0009

**Table 2:** IL-6 and TNF-α mRNA expression levels in precision-cut lung slices (PCLSs) untreated or treated for 4 h with lipopolysaccharide (LPS), unexposed or exposed to NIR laser protocols A, B, or C, with or without starvation, and analyzed 6 h after NIR laser treatment. In control PCLSs, mRNA expression was quantified at the corresponding timepoint (i.e., 2 h after the time at which laser treatment would have been applied). Means ± SDs.

**IL-6**										
**Controls**		**Protocol A**			**Protocol B**			**Protocol C**		
Untreated	LPS	Untreated	LPS	LPS starvation	Untreated	LPS	LPS starvation	Untreated	LPS	LPS starvation
0.05355 ± 0.01080	0.2334 ± 0.0329	N/A	0.1492 ± 0.1492	0.1362 ± 0.0091	N/A	0.1910 ± 0.0466	0.1447 ± 0.0120	0.0508 ± 0.0191	0.1979 ± 0.0128	N/A
**TNF-α**										
**Controls**		**Protocol A**			**Protocol B**			**Protocol C**		
Untreated	LPS	Untreated	LPS	LPS starvation	Untreated	LPS	LPS starvation	Untreated	LPS	LPS starvation
0.00952 ± 0.00071	0.0369 ± 0.0025	N/A	0.0263 ± 0.0028	0.0227 ± 0.0011	N/A	0.0245 ± 0.0035	0.0156 ± 0.0102	0.0041 ± 0.0038	0.0295 ± 0.0033	N/A

N/A: The value was not measured due to the poor quality of the isolated mRNA.

## Data Availability

The original contributions presented in this study are included in the article. Further inquiries can be directed to the corresponding author.

## Abbreviations

BPD	bronchopulmonary dysplasia
IL-1β	interkeukin-1β
IL-6	interkeukin-6
IL-10	interkeukin-10
IL-18	interleukin-18
LPS	lipopolysaccharide
MLS	multiwave locked system
NIR	near-infrared
NLRP10	nucleotide-binding domain leucine-rich repeat-containing receptor-10
PBMT	pulmonary photobiomodulation therapy
PCLSs	precision-cut lung slices
RT-qPCR	reverse transcription quantitative polymerase chain reaction
TNFα	tumor necrosis factor α
